# Application of the Chinese version of the addiction profile index (API) in drug users: an analysis of validity and measurement invariance across genders

**DOI:** 10.1186/s13011-020-00263-9

**Published:** 2020-04-09

**Authors:** Huiyuan Gao, Meizhu Liu, Xu Luo, Jun Zhang, Taisheng Cai

**Affiliations:** 1 Medical Psychological Center, The Second Xiangya Hospital, Central South University, Changsha, Hunan 410011 China; 2 Medical Psychological Institute of Central South University, Changsha, Hunan 410011 China; 3 National Clinical Research Center for Mental Disorders, Changsha, Hunan China; 4 Hunan Judicial Police Vocational College, Changsha, China

**Keywords:** Addiction profile index, Severity, Reliability, Validity, Measurement invariance

## Abstract

**Background:**

In China, substance use disorders represent a significant burden on public health and the economy. However, while the range of drugs and drug markets expands and diversifies, the instruments available to evaluate users’ dependence statuses from multiple dimensions have become insufficient. Accordingly, the present study presents the Chinese version of the Addiction Profile Index (API), explores its reliability and validity, and investigates the measurement invariance between males and females with substance use disorders.

**Methods:**

The API, a self-report questionnaire, was administered to 2252 people with substance use disorders who were undergoing treatment in compulsory detoxification institutions located in five provinces in China (943 females; mean age = 33.5 years old, *SD* = 8.6). Additionally, to ensure the authenticity of the collected data, the study’s volunteers completed the Drug Use Disorders Identification Test (DUDIT), DUDIT-Extended (DUDIT-E), and the Health Scale for Drug Abusers (HSDA).

**Results:**

The revised API, with its updated substance list, featured 34 items. The new four-factor model, incorporating behavioral symptoms of dependence, impact on social life, cravings, and motivations for detoxification, explained 55.30% of the total variance, indicating a good fit. Moreover, Cronbach’s α and mean item coefficient values showed good internal consistency reliability. Regarding criterion validity, the revised factors were moderately to highly correlated with their corresponding subscales in the DUDIT, DUDIT-E, and HSDA. In addition, the multigroup confirmatory factor analysis demonstrated that a measurement invariance of the revised four-factor model across genders was supported, fully assuming different degrees of invariance. The three factors of symptoms, social life, and motivation exhibited significant differences between male and female participants in the *t* test results (*p* < 0.01).

**Conclusions:**

The Chinese version of the API shows good psychometric properties in terms of reliability and validity, and exhibits measurement equivalence across the genders. Therefore, it could be used to comprehensively assess the severity of drug dependence in people with substance use disorders.

## Backgroud

Substance use disorder (SUD), characterized by a cluster of cognitive, behavioral, and physiological symptoms indicating that the individual continues to use the substance despite significant substance-related problems [1], is acknowledged to be a worldwide public health problem. By the end of 2018, around 2.40 million drug users were officially registered in China [2]; methamphetamine was the most commonly used drug, with around 56.1% of all registered drug users reporting “ice” (methamphetamine) use, followed by heroin and ketamine, the use of both of which has increased rapidly in recent years and accounts for about 39.6% of drug users. Influenced by globalization, there have been several changes seen in the varieties and structures of drug use in China. Apart from the dominant position of the new synthetic amphetamine-type stimulants (ATS) drugs, the polydrug use issue is becoming more prominent with each passing day. As the China National Narcotic Control Commission reported [3], the mixing of opioids and other drugs with synthetic ATS drugs is taking place with increasing regularity since the synthetic drugs entered the market, in order to enhance their dependence effects.

Responding to the country’s serious and complicated drug situation, the Chinese government has been exerting considerable efforts and implementing targeted strategies in order to try to control it, especially in terms of prevention and harm reduction [3–5]. Firstly, laws and regulations have been enacted or modified (e.g., the Anti-Drug Law, the Regulation on Drug Rehabilitation) to explicitly stipulate that people found using drugs will be detained for up to 15 days, and people with substance use dependency will be sent for rehabilitation. In addition, there have been reductions in the opium poppy supply from the “Gold Triangle” area, and the “5–14” drug source interception mechanism has been further improved to stop drugs entering China, especially in Yunnan Province and the Guangxi Zhuang Autonomous Region. Thirdly, a variety of health education activities have been organized for young people, including classes on drug prevention and drug education information disseminated through multiple forms such as television, the internet, community interviews, and newspapers. Regarding harm reduction strategies, outreach activities incorporating voluntary HIV tests and privacy protection, methadone maintenance treatments, and needle-syringe programs have been carried out.

In recent years, three types of treatment settings have become available for drug users in China: compulsory isolated rehabilitation centers, voluntary detoxification institutions, and community-based drug rehabilitation and treatment [6]. Under Chinese law, drug users who have refused to receive community-based rehabilitation, or have failed to maintain abstinence in the community, or have been arrested for suffering from a severe drug use disorder will be sent for two years to a compulsory isolated rehabilitation center managed by the Ministry of Justice. In the voluntary detoxification institutions run by the local departments of health, medical treatments including methadone maintenance treatment are provided for 7 to 30 days. The community-based drug rehabilitation and treatments, organized by local government and sub-district offices, undertake periodical inspections and provide job training and psychological treatment for three years to individuals released from the compulsory detoxification institutions.

However, the instruments currently used in such institutions are not suitable for evaluating the dependence severity of individuals’ drug use disorders. One issue is that the questionnaires’ evaluation of dependence severity is constrained by the drug types featured in the questionnaires. While new patterns of synthetic drug and polydrug use are becoming increasingly prevalent, the existing measurement instruments are mainly aimed at traditional substances (e.g., opioid drugs or alcohol, with the corresponding instruments including the Opioid Addiction Severity Inventory and the Penn Alcohol Craving Scale), and so are not suitable for being generalized to individuals dependent on the new synthetic drugs (e.g., ice, ketamine, etc.) or those who abuse multiple drug types [7–9]. Secondly, while some widely used instruments with multiple dimensions (e.g., the Addiction Severity Index) theoretically can be used for a comprehensive assessment, professional feedback from physicians is also needed, which takes so much time to implement that it becomes unworkable in actual clinical practice [10, 11]. Thirdly, due to limited access to or availability of some categories of participants, a number of the scales have been developed through reference to a small sample size or low numbers of female drug users [12], which means that their efficacy or applicability cannot be generalized to the total population as they are not sufficiently representative.

Endeavoring to address this gap, the Chinese version of Addiction Profile Index (API) was introduced and firstly developed with reference to a large sample of drug users, especially a representative proportion of female participants, thereby exhibiting greater representativeness in terms of sampling [13]. Moreover, the self-reporting scale takes a convenient amount of time to complete and has multiple dimensions, with dependence severity assessed comprehensively yet expediently. Lastly, referring to the results of the questionnaire, including an attached drug-use list, detoxification institutions will be better able to formulate the corresponding therapeutic schedules or management programs for drug users, no longer constrained by today’s increasingly diversified substance categories.

Findings from previous studies have indicated a significant difference between genders with respect to the severity of drug dependences, which could be related to differences in physical characteristics or social demands (e.g., social responsibility, social roles, etc.) [14, 15]. Equally, though, it is possible that the discrepancies observed between the genders might be a result of the design of the measurements themselves, rather than corresponding to actual differences between female and male drug users. Therefore, when evaluating dependence severity, it will be necessary to assess the measurement equivalence of the psychological scale across genders.

Overall, the present study examines the psychometric properties of the API in China and explores the measurement invariance across genders to provide more accurate information regarding respective dependence severities.

## Methods

### Participants

The participants in the current study were randomly recruited from nearly 33 compulsory detoxification institutions in five provinces in China (i.e., Guangdong, Shanxi, Sichuan, Guizhou, and Hunan). All participants with substance use disorders met the criteria of the Diagnostic and Statistical Manual of Mental Disorders (DSM-5) and the International Classification of Diseases (ICD-10). The exclusion criteria were: 1) participants were in the period of acute withdrawal; 2) participants were suffering from major mental disorders, infectious diseases, or some form of physical disability;3) age < 18 years; and 4) participants had a relatively low level of education and could not independently complete the test(≤grade 2 of primary school).

The final sample consisted of 2252 participants, including 58.13% (1309) men and 41.87% (943) women. No financial incentives were given to participants in our study. Even though it meant decreased workloads for those participants for the day—their salaries were paid as usual.

This study was approved by the Medical Ethics Committee of the Second Xiangya Hospital, Central South University (Changsha, China). Participation in this study was entirely voluntary to ensure the authenticity and validity of the collected data. All participants provided written informed consents prior to the initiation of the study.

### Measurements

#### API

The API, a self-reported questionnaire, was established by Ogel in 2012 [13] for assessing the severity of drug dependence. The original version consisted of 37 items divided into the following five dimensions: 1) 12 items on the characteristics of substance use (a drug-use list); 2) 8 items on the diagnosis of dependence; 3) 10 items on the effects of substance use on the user; 4) 4 items on craving; and 5) 3 items on motivation to quit using substances. In general, the API was a valid and reliable questionnaire.

#### Drug use disorders identification test (DUDIT)

This scale was developed by Berman in 2003 [16]. It consists of 11 items for screening individuals with drug problems from the following three aspects: symptoms of dependence, physical and psychological problems related to substance use, and frequency of substance use. The results obtained from individuals with severe dependence and healthy participants showed that the scale had a satisfactory reliability and validity.

#### DUDIT–extended (DUDIT-E)

The scale Evren et al. [17] developed consists of 41 items separated into four subscales: drug frequency (D), positive (P) negative (N), and treatment (T). The results of the reliability and validity analysis revealed that the scale exhibited good psychometric properties, which could be used for further and more detailed assessment of drug-related problems.

#### Health scale for Chinese drug abusers (HSDA)

This scale was developed by Cai in 2008 [18], and is used to comprehensively evaluate the physical and mental health of drug users. It consists of 120 items separated into the following 11 factors, the results of the validity and reliability analysis revealed that the scale exhibited satisfactory psychometric properties for the evaluation of people with drug use disorder in China.

### Procedure

With the authorization and approval of Ogel, a Chinese version of the API was developed using the Brislin standard back-translation technique [19]. Besides, a semi-structured interview [5, 20] was used in 24 voluntary participants with drug use disorder to explore the rationality of the items and enhance the suitability of each item for the actual drug use by Chinese users. Meanwhile, the investigators were trained on providing consistent instruction and attitude toward the participants to establish a standardized test. The purpose of the test was explained and the confidential nature of the study was emphasized during the test to improve participant cooperation and reliance.

Subsequently, the nationwide data were formally collected. All participants signed an informed consent form before the test began. After completion of the API, the DUDIT, DUDIT-E, and HSDA were administered to a proportion of users for the assessment of criterion-related validity. The whole process of the test was performed in silence, with suitable tables and chairs supplied, a pen with black ink, and spectacles as necessary. In addition, there were no more than 30 participants in each setting, and all the questionnaires were completed within 40 min.

### Data analysis

Double-entry and validation were performed with EpiData 3.1 to establish the database [21]. Confirmatory factor analysis (CFA) was conducted using the statistical software Mplus 7.0, while the SPSS version 21.0 software was used for other data analyses.

It was noted that the participants in the factor analysis were randomly divided into two equal groups: one for exploratory factor analysis (EFA) and another for CFA. Other validity analyses of the questionnaires were conducted in all participants. Data processing chiefly comprised the following steps.

- Step 1: Item analysis. The scores of the total API were ranked from the lowest to the highest in ascending order. Of the participants, 27% were at the top and the bottom of this ranking (i.e., high-score group and low-score group, respectively). An independent sample t-test was used to examine the difference between the high-score group and the low-score group [22].
- Step 2: EFA. Principal component factor analysis with orthogonal varimax rotation was used to extract the common factor in the first half of the sample (sample 1), with an eigenvalue of > 1.0 for factor extraction. Besides, items with a factor loading of 0.4 or greater were considered to contribute to the factor. PCA was implemented to all items of API which were treated as a single group and analyzed together, except for a drug-use list.
- Step 3: Reliability. Cronbach’s alphas (α) and mean inter-item correlations (MIC) were used to evaluate the internal reliability of the API. In general, the Cronbach’s α coefficients above 0.70 is considered acceptable but an α of 0.60 is also used [23]. An optimal range of 0.10–0.50 was set for the MIC; lower than 0.10 and it is unlikely that a single total score could adequately represent the complexity of the items; higher than 0.50 and the items on a scale to be overly redundant and the construct measured too specific [24]. The relationships between the total scale and the five facets were also examined using Pearson’s r.
- Step 4: Validity. a.) To analyze the construct validity, maximum likelihood estimation in CFA was used to confirm the new factorial structure model in the second split-half of the sample (sample 2). As a chi-square test is susceptible to the sample size, even a small difference would result in a significance as the sample size increases. Accordingly, there were three fit indices in the present study: the root mean square error of approximation (RMSEA), comparative fit index (CFI), and Tucker Lewis index (TLI) [25, 26]. The criteria together used to evaluate model fit were as follows: TLI ≥ 0.90, CFI ≥ 0.90, and RMSEA≤0.08. Meanwhile, the factorial structures of the original version and the Chinese version were compared, and the model that fitted the data better was chosen for the subsequent measurement invariance analysis. b.) Besides, criterion-related validity was assessed using the Pearson correlation coefficient, wherein validity is considered good if the value obtained is between 0.4 and 0.7 [27]. Therefore, the correlation coefficient of the API with its corresponding subscales in the DUDIT, DUDIT-E, and HSDA were calculated.
- Step 5: Measurement invariance. After identification of the best fitting API model, the measurement invariance across genders in the total sample was examined through multigroup CFA under the framework of the structural equation model from the following four degrees: configural invariance, weak factorial invariance, strong factorial invariance, and strict invariance. Furthermore, CFI and TLI differences were used to evaluate invariance across consecutive models: both ΔCFI ≤0.01 and ΔTLI ≤0.01 were considered evidence of invariance, as suggested by Cheung and Rensvold [28].

## Results

### Descriptive statistics and item analysis

The demographic information of the 2252 participants randomly recruited from the compulsory detoxification institutions may be summarized as follows: 1) there were 139 males for every 100 females; 2) the mean age was 33.52 years old, with a standard deviation of 8.6; 3) most of the participants were unemployed (about 47.29% of all participants); and 4) the substances mainly used were opioids and amphetamines. Other demographic information is illustrated in Table 1.

**Table 1:** Demographic data of the drug users in this study

	Number of participants (%)/Mean ± SD					
	Shanxi (*n* = 487)	Sichuan (*n* = 625)	Guizhou (*n* = 482)	Guangdong (*n* = 508)	Hunan (*n* = 150)	Total (*N* = 2252)
**Gender**						
Male	348 (26.6)	448 (34.2)	141 (10.8)	247 (18.9)	125 (9.5)	1309 (100)
Female	139 (14.7)	177 (18.8)	341 (36.2)	261 (27.7)	25 (2.7)	943 (100)
**Age**						33.52 ± 8.629
18–25	60 (13.5)	148 (33.3)	106 (23.9)	111 (25.0)	19 (4.3)	444 (100)
26–34	200 (23.3)	275 (32.1)	135 (15.8)	189 (22.1)	58 (6.8)	857 (100)
35–44	173 (26.2)	138 (20.9)	144 (21.8)	160 (24.2)	46 (7.0)	661 (100)
≥ 45	54 (18.6)	64 (22.1)	97 (33.4)	48 (16.6)	27 (9.3)	290 (100)
**Employment**						
Unemployed	195 (18.3)	286 (26.9)	288 (27.0)	221 (20.8)	75 (7.0)	1065 (100)
Employed	292 (24.6)	339 (28.6)	194 (16.3)	287 (24.2)	75 (6.3)	1187 (100)
**Educational level**						
≤ Primary	121 (26.7)	123 (27.1)	79 (17.4)	116 (25.6)	15 (3.3%)	454 (100)
Middle	233 (19.7)	328 (27.7)	262 (22.1)	289 (24.4)	73 (6.2)	1185 (100)
High	82 (18.2)	135 (29.9)	101 (22.4)	84 (18.6)	49 (10.9)	451 (100)
≥ University	31 (30.7)	31 (30.7)	21 (20.8)	10 (9.9)	8 (7.9)	101 (100)
**Category**^**a**^						
Opioids^b^	180 (17.4)	209 (20.3)	321 (31.1)	266 (25.8)	56 (5.4)	1032 (100)
Cannabis	33 (14.0)	102 (43.4)	22 (9.4)	60 (25.5)	18 (7.7)	235 (100)
ATS^c^	318 (19.6)	538 (33.2)	266 (16.5)	371 (22.9)	129 (8.0)	1622 (100)
Hallucinogen^d^	76 (15.4)	204 (42.4)	49 (10.2)	111 (23.1)	41 (8.5)	481 (100)
Pills-Medicine^e^	84 (52.2)	38 (23.6)	16 (9.9)	17 (10.6)	6 (3.7)	161 (100)
Others^f^	151 (51.0)	55 (18.6)	19 (6.4)	61 (20.6)	10 (3.4)	296 (100)

a: Usage in previous period

b: Opioids = Heroin + Opiates

c: ATS = Amphetamine-type stimulants = Ice + Magu +Ecstasy

d: Hallucinogen = Ketamine

e: Pills-Medicine = Dolantin +Morphine + Rohypnol +Rivotril +Akineton + Tantum

f: Others = GHB + LSD + Crack + Cocaine

Abbreviations: *SD* standard deviation

For the item analysis. An independent-sample t-test was utilized to explore the difference between the two groups (27%). The results showed that 25 items exhibited satisfactory discrimination among them.

### EFA

Principal component analysis with orthogonal varimax rotation was conducted to explore the best fitting factor model of the API in a random split-half of the whole sample (*n* = 1126). The results exhibited that the Kaiser–Meyer–Olkin was good (0.931) and Bartlett’s test of sphericity was statistically significant (*P* < 0.01), demonstrating suitability for the EFA [29]. Four factors were obtained on the basis of the normal extraction factor of the eigenvalue > 1.0, accounting for 55.30% of the total variance. As shown in Table 2, the results indicated that all 25-item loadings of the API to corresponding factors were > 0.40 in the current study. Furthermore, the first factor (10 items) indicated the behavioral symptoms of dependence reflecting the extrinsic behaviors of drug seeking. The second factor (9 items) was defined as the impact on social life embodying the impaired severity of social function among drug users. The third factor termed craving (3 items) was used to describe the subjective feeling of spiritual desire of users for drugs. Lastly, the fourth factor termed intention of detoxification (3 items) was interpreted as the intensity of motivation to quit using drugs.

**Table 2:** Factors and factor loadings of the Chinese and original versions of the API with EFA

Item	Ogel		Present	
	Factor^a^	Loadings	Factor^a^	Loadings
Tolerance^b^	1	.590	1	.771
Abstinence^b^	1	.494	1	.751
Low resistance to substance use	1	.413	1	.779
Frequent attempts to quit	1	.683	1	.703
Spending too much time to use	1	.642	1	.741
Discontinuing other activities	1	.577	1	.590
Family relationship problems	2	.742	2	.631
Career/educational problems	2	.676	2	.638
Physical health problems	2	.622	2	.678
Psychological problems	2	.729	2	.733
Economic problems	2	.461	2	.534
Problematic relationships with friends and neighbors	2	.372	2	.709
Getting into trouble with substance use	2	.676	2	.654
Legal problems	2	.672	2	.476
Substance use during daytime	2	.512	1	.546
Using the substance when you do not want to	3	.619	3	.643
Family and neighbors are worried about you	2	.372	2	.436
Thinking about substance use	3	.832	3	.699
Craving substance use	3	.871	3	.614
Difficulty controlling the substance use	3	.520	1	.608
Problems associated with substance use	4	.710	4	.611
Considering quitting or decreasing use	4	.767	4	.774
Importance of quitting or decreasing use	4	.769	4	.743
% of variance		52.3		55.299

Note: Tolerance = (Item 13+ Item 14)/2, Abstinence = (Item 15 + Item 16)/2

^a^Factor 1: diagnosis, factor 2: effects on person’s life, factor 3: craving, factor 4: motivation

^b^Factor 1: behavioral symptoms of dependence, factor 2: the impact on social life, factor 3: craving, factor 4: motivations of detoxification

Abbreviations: *API* Addiction Profile Index, *EFA* exploratory factor analysis

### Reliability

In the whole sample, the Cronbach’s alpha correlation coefficient for the total API was 0.914, and its four factors respectively were 0.889, 0.858, 0.555 and 0.690. The MIC of total API was 0.306, ranging from 0.268 (craving) and 0.482 (behavioral symptoms), see Table 4.

### Intercorrelation among the API and its four factors

Pearson correlation coefficients of the total score of the API and the four factors ranged from 0.582 to 0.852, see Table 4, and all correlation coefficients between factors were positive and statistically significant (*p* < 0.01) except for the facets of craving with motivation.

### Construct validity and criterion-related validity

As shown in Table 2, two of these 25 items of the revised API loadings on the corresponding factor were inconsistent with the original English version. Thus, the factorial structures of the model developed by Ogel and the newly obtained model were analyzed using CFA in the remaining half of the sample (*n* = 1126). The results showed that the new model achieved a satisfactory standard and provided the best fit for data on Chinese drug users (RMSEA ≤0.08, TLI ≥0.90, and CFI ≥0.09).

The results of the criterion-related validity showed that the correlation coefficient of the API with its corresponding subscales of other questionnaires ranged from 0.559 to 0.684, the moderate-to-high correlations were positive and significant(*p* < 0.01), see Table 4.

### The measurement equivalence across genders

We performed a multigroup CFA in the total sample to examine whether the scale exhibits measurement invariance across genders (Table 3). In the configural equivalence test, all parameters (i.e. any factor load), as well as the intercept of the observed variables and the residuals were allowed to be freely estimated. The following fitting indices were obtained: TLI = 0.911, CFI = 0.921, and RMSEA = 0.056. When the fitting indices fulfils the criteria of model fit (TLI ≥ 0.9, CFI ≥ 0.9, and RMSEA≤0.08), configural equivalence was established and the configural model was used as the baseline model for subsequent analysis. Equivalent factor loads (same load for the indicator at different measurement points) were set based on the baseline model to verify whether the same item represents the same concept across different groups. The weak equivalence test showed a ΔCFI of 0.002 and a ΔTLI of 0.001. The equivalent of the measurement intercept of each index was set based on the previous step. All fitting indices met the fit criteria: ΔCFI and ΔTLI were 0.01 and 0.007, respectively. Lastly, the error variance equivalent was set on the basis of the third step. The fitting indices suggested that the model can be fitted with a ΔCFI and ΔTLI equal to 0.006 and 0.002, respectively. These results support the assumption that the criterion error variance is equivalent and the strict equivalence equivalent holds.

**Table 3:** Confirmatory factor analysis of the Addiction Profile Index

Model	χ^2^	df	p	CFI	TLI	AIC	BIC	RMSEA	SRMR		ΔCFI	ΔTLI
Model-Ogel	1523.781	224	<.01	.874	.858	74,446.315	74,823.297	.072	.079		–	–
Model-Present	1088.710	224	<.01	.916	.905	73,964.001	74,340.983	.059	.048		–	–
Model A	1774.386	448	<.01	.921	.911	123,603.732	124,435.064	.056	.047		–	–
Model B	1823.518	467	<.01	.919	.912	123,604.962	124,330.992	.056	.051	B vs. A	−.002	.001
Model C	2012.815	486	<.01	.909	.905	123,769.823	124,390.551	.058	.053	C vs. B	−.01	−.007
Model D	2142.319	509	<.01	.903	.903	123,881.854	124,375.111	.058	.057	D vs. C	−.006	−.002

Notes: Model A, configural invariance; Model B, metric invariance; Model C, scalar invariance; Model D, strict invariance

Abbreviations: *df* degrees of freedom, *TLI* Tucker Lewis Index, *CFI* comparative fit index, *RMSEA* root mean square error of approximation, *SRMR* standardized root mean squared residual, *BIC* Bayesian information criterion, *AIC* Akaike information criterion

### The differences in four factors between genders

The t-test was used to compare the differences in four factors of the revised scale across genders among all participants. The results are exhibited in Table 4. Firstly, the results of the independent sample t-test showed that there was no significant difference between the two genders in the score of both the total scale and the craving factor. However, in terms of the behavioral symptoms of the dependence factor, the impact on the social life factor, and the motivation of the detoxification factor, the significant differences were obtained in our study (Table 5).

**Table 4:** The difference of the API across genders and the correlation coefficient of factors

	API						
	n	Substance	Symptoms	Social life	Craving	Motivation	Total
Substance		–					
Symptoms		.427**	–				
Social life		.409**	.681**	–			
Craving		.350**	.418**	.330**	–		
Motivation		.128**	.347**	.483**	.025	–	
Total		.582**	.839**	.852**	.567**	.629**	–
Mean ± SD		3.36 ± 1.07	23.69 ± 7.69	27.87 ± 7.93	5.45 ± 2.21	10.83 ± 2.90	13.16 ± 2.89
**DUDIT**	240						
Total			**–**	–	–	–	.633
Dependence			.684	–	–	–	–
**DUDIT-E**	240						–
Negative (N)			**–**	.663	–	–	–
Treatment (T)			–	–	–	.559	–
**HSDA**	240						
Craving			–	–	.560	–	–
**Cronbach’s α**	2252		.889	.858	.555	.690	.914
**MIC**	2252		.482	.403	.268	.426	.306

^**^Correlation is significant at the 0.01 level (two-tailed)

Abbreviations: *API* Addiction Profile Index, *DUDIT* Drug Use Disorders Identification Test, *DUDIT-E* Drug Use Disorders Identification Test–Extended, *HSDA* Health scale for Chinese Drug Abuser, *SD* standard deviation

**Table 5:** Scores on total API and each of factor separated by gender (Means ± SD)

	Male (***n*** = 1309)	Female (***n*** = 943)	t	p
Symptoms	23.19 ± 7.70	24.29 ± 7.73	−3.108**	.002
Social life	28.28 ± 8.09	27.19 ± 7.78	2.962**	.003
Craving	5.46 ± 2.24	5.48 ± 2.25	−0.18	.857
Motivation	10.69 ± 2.92	10.99 ± 2.84	−2.237**	.025
Total	13.17 ± 2.97	13.27 ± 2.88	−0.728	.467

^**^Correlation is significant at the 0.01 level (two tailed)

## Discussion

In current study, we introduced the API. Apart from the analysis of reliability and validity of the Chinese version, the measurement invariance across genders was examined among 2252 drug users.

The 12 items for the addictive substance included information regarding the substances used and the frequency of use, and was termed the characteristics of substance use. According to the major ingredients of a drug as well as the proportion of the population using that drug, the results of demographic data revealed that substances “Magu,” and “Ketamine” should be the new additions. And two items (i.e., “Alcohol” and “Volatile substances”) were deleted as the scale primarily targeted drug users. This classification is similar to that stated in the Diagnostic and Statistical Manual of Mental Disorders, more importantly, it is closely linked to the characteristics of the Chinese drug market [2]. Hence, it is valuable for further research on drug dependence (for further details, see the appendix).

The EFA in the first split-half of the sample showed that the factor of behavioral symptoms of dependence contains two additional items in the present version versus the original version. One of the two items is “Have you ever used (substance) during daytime?”, which belonged to the factor of the effect of substance use on everyday life in the original version. The reason for this difference may be the larger proportion of unemployed participants included in the current study (47.29%). There was no obvious influence on the daily life or work efficiency of the participants even if the drug using occurred during daytime. On the contrary, the level of drug use throughout the day could be used to assess the severity of dependence in individuals in clinical settings [30]. The second item was “Was it difficult for you to resist using (substance) when it was available?”, belonging to the factor of craving in the original version. The factor of craving, also termed psychological craving [31], was described as a subjective feeling from within regarding one’s desire to use a particular substance. From this perspective, we found that the information collected from the three items of the craving factor in the present version basically covered the content of the above definition, such as being reminded of the feeling of pleasure with drug use. However, the factor of behavioral symptoms of dependence reflected a judgement of dependence severity based on individual extrinsically behavioral performance. Furthermore, some researches [32, 33] put forward the notion that different languages and cultural factors could produce distinct factor structures of the scale. From this perspective, the above differentiation of factor structures may be closely related to the linguistic translation of the scale, particularly from the original English to the Chinese version, where semantic and cultural differences in understanding may be inevitable.

Cronbach’s α coefficient was used to verify the internal consistency reliability of the API. DeVellis [23] suggested that a Cronbach’s α coefficient > 0.60 is also considered acceptable. From this perspective, the Cronbach’s α coefficient for the total API and for the three other subscales, except craving, showed a highly significant correlation, reflecting good internal consistency. Regarding the craving factor, the results of the relatively lower alpha coefficient might be related to the relatively fewer items included in this factor [34]. According to Briggs and Nenue [24], the mean inter-item correlation (MIC) was used for reliability estimate in that it is not influenced by scale length, and an optimal range of 0.10–0.50 was set for MIC. Hence, all the mean inter-item coefficients were above the lowest accepted level which also supports the good internal consistency of the API.

For the intercorrelation, Pearson correlation coefficients between the total API and its factors indicated that the validity of the scale is high. Besides, the correlation coefficients between each factor were relatively week. This is consistent with the characteristics of the scale which could independently reflect the different aspects of dependence, provided further support for the validity of the API as a comprehensive measure of drug use experiences.

For criterion-related validity, the results demonstrated that the revised API reaches satisfactory standards (r_p_ = 0.4–0.7) by comparing the scores of four factors and the total API with their corresponding subscales of other questionnaires. Especially, both of the results for the social life and the motivation factor are consistent with the findings reported by Evren in Turkey [17].

Compared with the original version, a single group of CFA showed that the new model fitted the data better among Chinese drug users, which powerfully supported the revised version of the API could be used as the baseline model for subsequent analyses of measurement invariance across genders. Besides, the findings of multigroup CFA indicated the observed scores of the Chinese version of the API have the same meaning across genders, demonstrating that the factor loadings, intercepts, and error variances of each of the items of the revised API are equal, achieving complete equivalence among Chinese drug users. In other words, male and female drug users would respond to each item of the API in a similar manner, and their obtained scores could be directly compared between genders.

Furthermore, we examined the differences between the study’s male and female participants with SUD in terms of their respective scores for the total API as well as its factors, partly following a prior study Diaz-Mesa et al. performed [35]. The significant differences, in respect of behavioral symptoms, social life, and motivation for detoxification, may be associated with gender-determined relative behavioral inhibitory control of the executive function system [36], social roles and responsibilities [37], and personality characteristics [38].

## Conclusions

The Chinese version of the Addiction Profile Index shows good psychometric properties in terms of reliability and validity, which could be used to comprehensively assess the severity of dependence in drug users. Furthermore, the measurement invariance and factorial structures of the questionnaire provided more accurate information of gender difference for reference.

## Data Availability

All data and materials related to the study can be obtained by contacting the first author at caitaisheng@csu.edu.cn.

## Appendix

**Table 6:** Instruction: please tick the appropriate box according to the actuality of drug using as before. (√)

	Only a few times	1–3 times a month	1–5 times a week	Almost everyday
**1 Opioids** (Heroin, opiates)				
**Amphetamine-type stimulants**				
2 Ice				
3 Magu				
4 Ecstasy				
**5 Cannabis**				
**6 Hallucinogen** (Ketamine, etc.)				
**7 Pills-Medicine** (calming/sleeping, pain-relievers, etc.)				
**8 Others** (LSD,GHB, Cocaine, Crack, etc.)				

9 How often do you experience problems due to the effect of the (substance) (i.e. loss of consciousness, overdose, loss of control, etc.)

[1] Never [2] Rarely [3] Sometimes [4] Usually Almost always

## Abbreviations

API: Addiction Profile Index
CFA: Confirmatory factor analysis
CFI: Comparative Fit Index
DUDIT: Drug Use Disorders Identification Test
DUDIT-E: Drug Use Disorders Identification Test-Extended
EFA: Exploratory factor analysis
HSDA: Health Scale for Drug Abuser
MIC: Mean inter-item correlations
RMSEA: Root mean square error of approximation
SRMR: Standardized root mean squared residual
TLI: Tucker Lewis Index

## References

[1] American Psychiatric Association (2013). Diagnostic and statistical manual of mental disorders (DSM-5®): American Psychiatric Pub.

[2] China Nation Narcotic Control Commission (2019). 2018 Annual Report on Drug Control in China.

[3] H-q S, Bao Y-p, S-j Z, Meng S-q LL (2014). The new pattern of drug abuse in China. Curr Opin Psychiatry.

[4] Fang YX, Wang YB, Shi J, Liu ZM, Lu L. Recent trends in drug abuse in China. Acta Pharmacol Sin. 2006. 10.1111/j.1745-7254.2006.00270.x 16412261

[5] Yang M, Mamy J, Gao P, Xiao S, Li S. From Abstinence to Relapse: A preliminary Qualitative Study of Drug Users in a Compulsory Drug Rehabilitation Center in Changsha, China. PLOS ONE. 2015;10(6):e0130711. 10.1371/journal.pone.0130711 PMC4481107 26107639

[6] Zhao C, Liu Z, Zhao D, Liu Y, Liang J, Tang Y (2004). Drug abuse in China. Ann N Y Acad Sci.

[7] Flannery BA, Volpicelli JR, Pettinati HM. Psychometric Properties of the Penn Alcohol Craving Scale. Alcoholism: Clinical and Experimental Research. 1999;23(8):1289–95. 10470970

[8] Lian Z, Liu Z (2004). Reliability and development of Opiate Addiction Severity Chinese Journal of Drug Abuse Prevention and Treatment.

[9] WANG W, TANG J, LIU B (2010). The reliability and validity of Penn alcohol craving scale (PACS). J Psychiatry.

[10] Mclellan AT, Kushner H, Metzger D, Peters R, Smith I, Grissom G (1992). The fifth edition of the addiction severity index. J Subst Abus Treat.

[11] Luo W, Wu Z, Wei X, Jia W, Zhang Q, Li L (2007). Evaluation on responsibility of the Chinese version of addiction severity index. Mod Prev Med.

[12] United Nations Office on Drugs. World Drug Report 2018 (Sales No. E. 18. XI. 9). 2018.

[13] Ögel K, Evren C, Karadağ F, Gürol DT. The development, validity, and reliability of the Addiction Profile Index (API). Turk Psikiyatri Dergisi. 2012;23(4):263–75. 23225127

[14] Grella CE, Lovinger K (2012). Gender differences in physical and mental health outcomes among an aging cohort of individuals with a history of heroin dependence. Addict Behav.

[15] Lynch WJ, Roth ME, Carroll ME (2002). Biological basis of sex differences in drug abuse: preclinical and clinical studies. Psychopharmacology.

[16] Berman AH, Bergman HT, Schlyter F (2005). Evaluation of the drug use disorders identification test (DUDIT) in criminal justice and detoxification settings and in a Swedish population sample. Eur Addict Res.

[17] Evren C, Umut G, Bozkurt M, Evren B, Yilmaz H (2017). Psychometric properties of the Turkish version of drug use disorders identification test–extended (Turkish DUDIT-E) in substance-dependent adults under probation. Psychiatry Clin Psychopharmacol.

[18] Taisheng C, Guo J. The Health Scale for Chinese Drug Abuser (HSDA): Development, Reliability and Validity. Chin Mental Health J. 2007;21(11):762–7.

[19] Brinkmann C, Brixius K (2017). Erratum to: Peroxiredoxins and sports: new insights on the antioxidative defense. J Physiol Sci.

[20] Wang Z, Liu Z. Characteristics of addiction behaviors among methamphetamine dependent users: a qualitive research. Chin J Drug Depend. 2015;24(1):60–65.

[21] Paulsen A, Overgaard S, Lauritsen JM (2012). Quality of data entry using single entry, double entry and automated forms processing–an example based on a study of patient-reported outcomes. PLoS One.

[22] Diederich PB (2010). SHORTCUT ITEM-TEST CORRELATIONS FOR TEACHER-MADE TESTS. J Educ Meas.

[23] Devellis RF (1991). Scale development: theory and applications.

[24] Briggs SR, Nenue CJ (1986). The role of factor analysis in the development and evaluation of personality scales. J Pers.

[25] Hu LT, Bentler PM (1995). Evaluating model fit. Structural Equation Modelling Concepts Issues & Applications.

[26] Kline RB (2015). Principles and practice of structural equation modeling: Guilford publications.

[27] JQ F. Medical statistics and computer experiments. Shanghai: Shanghai Scientific and Technical Publishers; 2006. 440 p.

[28] Cheung GW, Rensvold RB. Evaluating Goodness-of-Fit Indexes for Testing Measurement Invariance. Struct Equ Model. 2002;9(2):233–55.

[29] Tobias S, Carlson JE (1969). BRIEF REPORT: BARTLETT'S test of SPHERICITY and chance findings in factor analysis. Multivar Behav Res.

[30] Kervran C, Fatséas M, Serre F, Taillard J, Beltran V, Leboucher J (2015). Association between morningness/eveningness, addiction severity and psychiatric disorders among individuals with addictions. Psychiatry Res.

[31] Shao C, Jiang K, Zhao M, Xu Y, Lu G, Xu H (2005). The gender differences of craving reaction and related indexes elicited by cues in heroin addicts. Chin J Drug Depend.

[32] Menold N, Kaczmirek L, Lenzner T, Neusar A (2014). How do respondents attend to verbal labels in rating scales?. Field Methods.

[33] Kim G, Decoster J, Huang CH, Chiriboga DA (2011). Race/ethnicity and the factor structure of the Center for Epidemiologic Studies Depression Scale: a meta-analysis. Cultur Divers Ethnic Minor Psychol.

[34] Peterson RA (1994). A meta-analysis of Cronbach's coefficient alpha. J Consum Res.

[35] Sanvicente-Vieira B, Rovaris DL, Ornell F, Sordi A, Rothmann LM, Niederauer JPO, Schuch JB, von Diemen L, Kessler FHP, Grassi-Oliveira R, Lima Santana G. Sex-based differences in multidimensional clinical assessments of early-abstinence crack cocaine users. PLOS ONE. 2019;14(6):e0218334. 10.1371/journal.pone.0218334 PMC6588218 31226126

[36] Li CSR, Cong H, Constable RT, Sinha R (2006). Gender differences in the neural correlates of response inhibition during a stop signal task. Neuroimage.

[37] Lash SJ, Copenhaver MM, Eisler RM (1998). Masculine gender role stress and substance abuse among substance dependent males. J Gend Cult Health.

[38] Vigna-Taglianti FD, Burroni P, Mathis F, Versino E, Beccaria F, Rotelli M (2016). Gender differences in heroin addiction and treatment: results from the VEdeTTE cohort. Subst Use Misuse.

